# Clinical outcome assessments for LBD trials – Should we borrow?

**DOI:** 10.1002/alz.091421

**Published:** 2025-01-09

**Authors:** Brad F. Boeve

**Affiliations:** ^1^ Department of Neurology, Mayo Clinic, Rochester, MN USA

## Abstract

Clinical outcome assessments (COAs) are an integral part of clinical trials. A fit‐for‐purpose COA with well‐selected endpoints can help determine the efficacy of a therapeutic intervention in the condition studied. The selection of the appropriate outcome measures depends not only on the condition but also on the disease stage and type of intervention studied.

In Alzheimer’s disease (AD) the clinical benefit of anti‐amyloid therapies has been assessed using outcomes that are specifically designed to measure the progression of Alzheimer’s disease during the early stages of the disease.

The enthusiasm to develop similar studies in Lewy body dementia (LBD) needs to be tempered with caution as there is no COA LBD that fully captures the progression of LBD, full symptom array, or impact of these upon function. This limitation has been partly met by the reliance on COAs developed for Alzheimer’s or Parkinson’s disease. However, these may not be valid or sensitive to treatment changes in LBD.

The impact of LBD on other domains beyond cognition including behavior, movement, and sleep represents one of the major challenges when considering the development of an LBD‐specific scale that encapsulates the severity of the disease as a whole.

During this presentation, we will discuss the limitations of the current outcome measures in LBD trials and what those developing a clinical trial focused on LBD in the near future need to be taken into consideration when deciding on a COA for disease‐modifying and symptomatic trials. We will also report on the current efforts to develop the Lewy Body Dementia‐Domain Rating Scale (LBD‐DRS), an LBD‐specific scale to be utilized in disease‐modifying therapy trials.

